# Identification of *Helicobacter pylori* Membrane Proteins Using Sequence-Based Features

**DOI:** 10.1155/2022/7493834

**Published:** 2022-01-12

**Authors:** Mujiexin Liu, Hui Chen, Dong Gao, Cai-Yi Ma, Zhao-Yue Zhang

**Affiliations:** ^1^Ineye Hospital of Chengdu University of TCM, Chengdu University of TCM, Chengdu 610084, China; ^2^School of Healthcare Technology, Chengdu Neusoft University, 611844 Chengdu, China; ^3^School of Life Science and Technology and Center for Informational Biology, University of Electronic Science and Technology of China, Chengdu 610054, China

## Abstract

*Helicobacter pylori* (*H. pylori*) is the most common risk factor for gastric cancer worldwide. The membrane proteins of the *H. pylori* are involved in bacterial adherence and play a vital role in the field of drug discovery. Thus, an accurate and cost-effective computational model is needed to predict the uncharacterized membrane proteins of *H. pylori*. In this study, a reliable benchmark dataset consisted of 114 membrane and 219 nonmembrane proteins was constructed based on UniProt. A support vector machine- (SVM-) based model was developed for discriminating *H. pylori* membrane proteins from nonmembrane proteins by using sequence information. Cross-validation showed that our method achieved good performance with an accuracy of 91.29%. It is anticipated that the proposed model will be useful for the annotation of *H. pylori* membrane proteins and the development of new anti-*H. pylori* agents.

## 1. Introduction

*Helicobacter pylori* (*H. pylori*) is a Gram-negative spiral-shaped bacterium that infects half of the human population worldwide. *H. pylori* causes gastric mucosa damage, chronic inflammation, and dysregulation of the gut community, increasing the risk of gastric cancer [1–3]. Attachment to the gastric mucosa is the first step in establishing bacterial colonization [4]. *H. pylori* membrane proteins such as antigen-binding adhesin (BabA), sialic acid-binding adhesin (SabA), outer inflammatory protein (OipA), and outer membrane protein Q (HopQ) can act as putative virulence factors that mediate the host-pathogen interactions, induce the release of inflammatory cytokines, and enhance the virulence property of the bacterium [4–6]. Thus, the identification of *H. pylori* membrane protein receptors contributes to the design of therapeutic drugs and vaccine development [7, 8].

**Figure 1:**
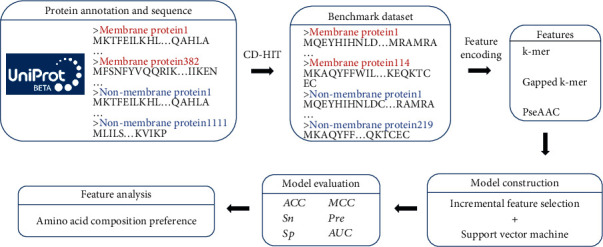
The workflow diagram of developing the *H. pylori* membrane protein prediction model.

Although *H. pylori* membrane proteins play a key role in attachment to and entry into host cells, only few have been described so far. There are some efforts in the prediction of membrane proteins [9, 10] for other germs like *Mycobacterial* [11] and *Chlamydiae* [12]. However, there are no machine learning-based approaches for the prediction of the *H. pylori* membrane proteins. In this study, we developed a comprehensive in silico approach for discriminating novel *H. pylori* membrane proteins using amino acid sequence-based criteria. First, the benchmark dataset was constructed based on a reliable source. Second, sequence-based feature encoding methods were used to represent protein sequences. Next, the incremental feature selection (IFS) technique with multiple feature ranking methods was applied to obtain the optimal feature set. Finally, a membrane protein prediction model was established based on the optimal feature set. The workflow can be seen in Figure 1.

## 2. Materials and Methods

### 2.1. Benchmark Dataset

An objective and strict benchmark dataset is fundamental for a robust prediction model construction [13–18]. The Universal Protein Resource (UniProt) [19] is a comprehensive resource for proteins and can be freely accessed at https://www.uniprot.org/. The 382 *H. pylori* membrane protein sequences and 1111 nonmembrane protein sequences were obtained from the UniProt. If a sequence contains nonstandard letters, the sequence was removed from the dataset. To avoid the influence of sequence similarity [20], CD-HIT [21] with 0.3 sequence identity was used to exclude highly similar membrane proteins. Finally, 114 (29.8% of the original) membrane proteins and 219 (19.7% of the original) nonmembrane proteins remained in the benchmark dataset.

### 2.2. Feature Encoding

Generally, feature encoding plays a crucial role for machine learning in model construction [22–28]. The feature encoding method determines the degree of sequence information mining. In this work, *k*-mer amino acid composition [29–31], gapped *k*-mer method [32], and pseudo-amino acid composition (PseAAC) [33–39] were used to formulate sequences.

Let the protein **S** be expressed as follows:

(1) $$\begin{array}{c} S=R_{1}R_{2}R_{3}R_{4}R_{5}\cdots R_{i}R_{i+1}\cdots R_{L}, \end{array}$$

where *L* denotes the length of the protein sequence and *R*_*i*_ is the *i*-th amino acid.

By using *k*-mer amino acid composition, a primary protein sequence **S** can be transferred into a vector **V**_*k*_ with 20^*k*^ elements according to the following formula:

(2) $$\begin{array}{c} V_{k}={\left[f_{1}^{k-\text{mer}}\;f_{2}^{k-\text{mer}}∙∙∙f_{i}^{k-\text{mer}}∙∙∙f_{20^{k}}^{k-\text{mer}}\right]}^{T}, \end{array}$$

where the symbol **T** means the transposition of a vector and *f*_*i*_^*k*−mer^ is the normalized frequency of the *i*-th *k*-mer amino acid component occurring in **S** and can be calculated by

(3) $$\begin{array}{c} f_{i}^{k-\text{mer}}=\frac{n_{i}}{\sum_{i=1}^{20^{k}}n_{i}}=\frac{n_{i}}{L-k+1}, \end{array}$$

where *n*_*i*_ means the number of occurrences of the *i*-th *k*-mer amino acid component in the sequence **S**.

With the increase of *k*, one protein sequence may have many *k*-mers absent, and its feature vector will contain a large number of zero values. To overcome this sparse problem, gapped *k*-mer (*k*-mer with *g* gap) was used. For example, “GG” with 3 gaps constitute the patterns “GNNNG,” where *N* represent any kind of amino acid. By using the gapped *k*-mer method, a primary protein sequence **S** can be transferred into a vector **V**_*g*_ with 20^*k*−*g*^ elements according to the following formula:

(4) $$\begin{array}{c} V_{g}={\left[f_{1}^{gk-\text{mer}}\;f_{2}^{gk-\text{mer}}∙∙∙f_{i}^{gk-\text{mer}}∙∙∙f_{20^{k-g}}^{gk-\text{mer}}\right]}^{T}, \end{array}$$

where the *f*_*i*_^*gk*−mer^ is the normalized frequency of the *i*-th *k*-mer with *g* gap amino acid component occurring in **S**.

PseAAC can represent a protein sequence in a discrete model without completely losing its sequence-order information. A primary protein sequence **S** can be transferred into a vector **V**_*p*_ with PseAAC according to the following formula:

(5) $$\begin{array}{c} V_{p}={\left[x_{1}\cdots x_{20}\;x_{20+1}\cdots x_{20+\lambda }\right]}^{T}, \end{array}$$

(6) $$\begin{array}{c} x_{i}=\left\{\begin{array}{cc} \frac{f_{i}}{\sum_{i=1}^{20}f_{i}+\omega \sum_{j=1}^{\lambda }\Theta_{j}}, & 1\leq i\leq 20,\\ \frac{\omega \Theta i-20}{\sum_{i=1}^{20}f_{i}+\omega \sum_{j=1}^{\lambda }\Theta_{j}}, & 20+1\leq i\leq 20+\lambda , \end{array}\right. \end{array}$$

where *f*_*i*_ is the normalized frequency of *i*-th amino acid, and Θ_*j*_ is the *j*-th sequence correlation factor that can be calculated by the product of the six physicochemical property numerical values between amino acids at different positions. *ω* is the weight factor for short range and long range.

### 2.3. Feature Selection and Modeling

To exclude noise and improve computational efficiency, feature selection is an indispensable step [23, 40–45]. Binomial distribution is one of the wonderful feature selection techniques that have been successfully applied in many works [46–48]. The high binomial distribution score indicates that the presence of the *k*-mer amino acid in a membrane protein sequence is not accidental. Analysis of variance (ANOVA) tests the ratio of the variance between groups and the variance within the groups to analyse the differences among group means [30]. The high ANOVA score means there is a big feature difference between the membrane protein group and the nonmembrane protein group. In this study, binomial distribution was used on *k*-mer features, and ANOVA was used on gapped *k*-mer and PseAAC features to winnow out the irrelevant features. Then, ANOVA was used to reprune all the redundant features.

After ranking the features according to their statistical scores, the IFS strategy with support vector machine (SVM) was adopted to determine the optimal feature set [49–53]. SVM is a classification algorithm that finds the optimal classification hyperplane in the high-dimensional feature space. The IFS strategy added features one by one to the feature set from a higher-ranked to a lower-ranked score. Once a new feature set was composed, LIBSVM [54] with 5-fold cross-validation was performed to train and test prediction models. The optimal feature set is defined based on the principle that the prediction model based on such features could achieve maximum accuracy. Finally, an SVM model was constructed based on the optimal feature subset for the membrane protein prediction.

### 2.4. Performance Evaluation Metrics

In order to assess the capability of the binary prediction method, six indexes, namely, accuracy (*ACC*), sensitivity (*Sn*), specificity (*Sp*), precision (*Pre*), Matthew's correlation coefficient (*MCC*), and the area under the receiver operating characteristic curve (AUC) [55–60], were used and formulated as

(7) $$\begin{array}{c} ACC=\frac{TP+TN}{TP+TN+FP+FN}, \end{array}$$

(8) $$\begin{array}{c} Sn=\frac{TP}{TP+FN}, \end{array}$$

(9) $$\begin{array}{c} Sp=\frac{TN}{TN+FP}, \end{array}$$

(10) $$\begin{array}{c} Pre=\frac{TP}{TP+FP\;}, \end{array}$$

(11) $$\begin{array}{c} MCC=\frac{TP\times TN-FP\times FN}{\sqrt{\left(TP+FN\right)\left(TP+FP\right)\left(TN+FP\right)\left(TN+FN\right)}}, \end{array}$$

where *TP* (true positive) and *TN* (true negative) present the numbers of correctly identified membrane proteins and nonmembrane proteins, respectively. *FP* (false positive) and *FN* (false negative) denote the number of nonmembrane proteins incorrectly classified as membrane proteins and the number of membrane proteins incorrectly classified as nonmembrane proteins, respectively. Receiver operating characteristics (ROC) analysis was used to measure the performance of the model with the varying decision thresholds [61–63]. Due to the small sample size, the result of the 5-fold cross-validation was used to evaluate the model performance.

## 3. Results and Discussion

### 3.1. Feature Optimization

**Figure 2:**
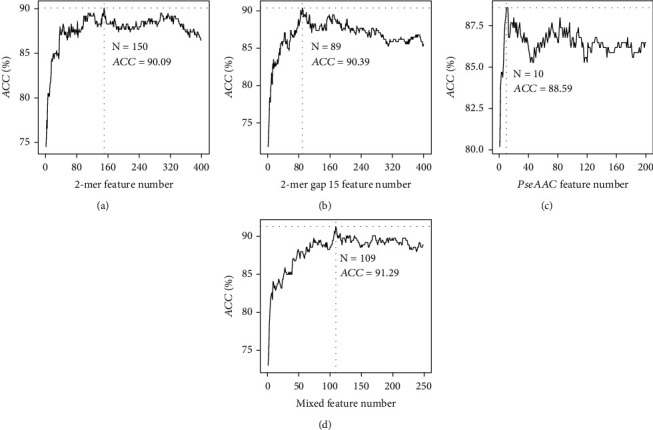
The IFS curves for (a) 2-mer features, (b) gapped 2-mer features, (c) PseAAC features, and (d) merged features.

As shown in equations (3), (4), and (5), the description of the protein sequences depends on parameters *k*, *g*, *ω*, and *λ*. For *k*-mer feature encoding, *k* = 2, 3, 4 was tried in this study. The model achieved the best accuracy of 90.09% with the top 150 binomial distribution-ranked 2-mer features (Figure 2(a)). For gapped *k*-mer feature encoding, we set *k* = 2 and traverse *g* from 1 to 20, when *g* = 15, and the model achieved the best accuracy of 90.39% with the top 89 ANOVA-ranked features (Figure 2(b)). For PseAAC, we set the weight factor *ω* = 0.5 and parameter *λ* from 1 to 70 with step size 5, and the best performance achieved was 88.59% when the *λ* is 20 and feature number is 10 (Figure 2(c)). To represent the sequence information comprehensively, all best feature subsets were merged and ranked by ANOVA. IFS was performed again to filter out the redundant features. As we can see in Figure 2(d), the model achieved the best accuracy of 91.29% when the top 109 ANOVA-ranked features were used to train the model.

### 3.2. Model Construction and Evaluation

Finally, 109 features were used to construct the SVM-based model for the prediction of membrane proteins. And the soft margin SVM penalty coefficient *c* and Gaussian kernel function width parameter *γ* are 0.5.

**Figure 3:**
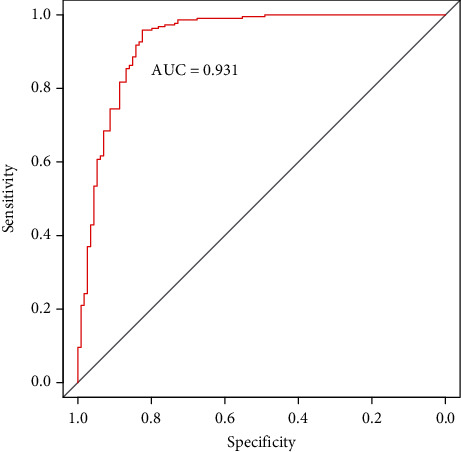
The ROC curves of the 5-fold cross-validation test.

To show the prediction capability of the final model, six evaluation metrics were calculated based on the result of the 5-fold cross-validation. The model achieved the  *ACC* of 91.29%, *Sn* of 82.46%, *Sp* of 95.9%, *Pre* of 91.26%, and *MCC* of 0.804. We also drew the ROC curve in Figure 3. It shows that the AUC reaches the value of 0.931, suggesting that the proposed model has an excellent prediction capability on membrane protein classification.

### 3.3. Amino Acid Composition (AAC) of Optimal Features

**Figure 4:**
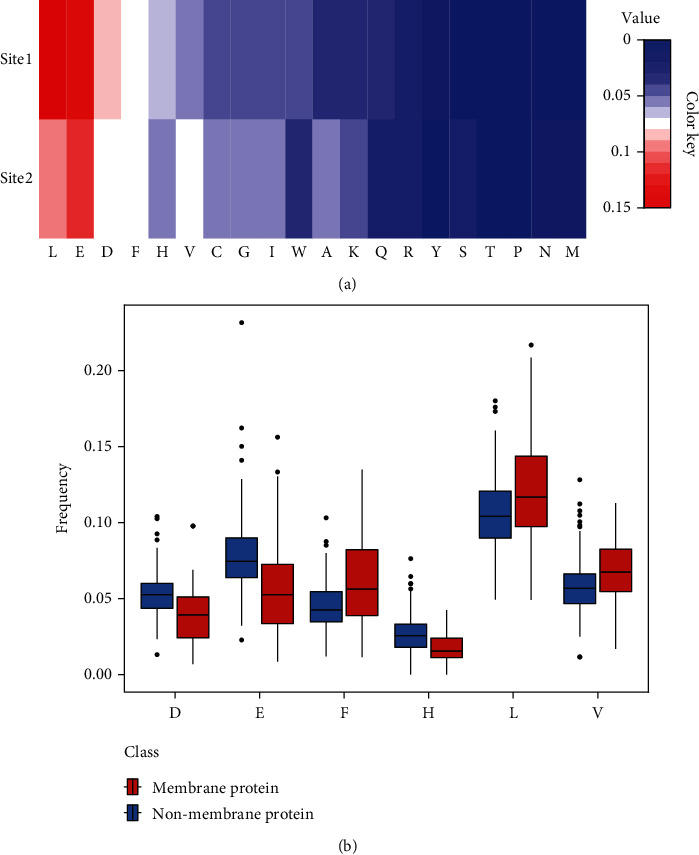
(a) The heat map of AAC of the model features. (b) The frequency of the six amino acids in the two classes.

The AAC of the model features was used to analyse the preference of membrane proteins for specific amino acids. Among the optimal feature set, there are 83 2-mer features, 16 gapped 2-mer features, and 10 PseAAC features. Focusing on the 2-mer and gapped 2-mer features, we found that the occurrence of leucine (L), glutamic acid (E), aspartic acid (D), phenylalanine (F), valine (V), and histidine (H) exceeds 50% of the total (Figure 4(a)). And the frequencies of F, L, and V in membrane protein sequences are significantly higher than those in nonmembrane protein sequences (*p* < 0.001). In contrast, the frequencies of D, E, and H in nonmembrane protein sequences are significantly higher than those in membrane proteins (*p* < 0.001) (Figure 4(b)).

## 4. Conclusions

*H. pylori* membrane proteins are an important class of molecules that play key roles in host-pathogen interactions. However, it is a new area in the prediction of *H. pylori* membrane proteins with machine learning methods. Hence, we developed an *H. pylori* membrane proteins predictor on the basis of sequence-based information. The model will powerfully support the discovery of *H. pylori* membrane proteins and the research of *H. pylori* infection. It has the potential to be significant in novel vaccine candidate antigens and drug development [64, 65]. In the future, we will stay focused on the *H. pylori* membrane protein prediction issues and screen the possible vaccine candidates and drug targets. Moreover, we will collect more data to train a deep learning model [66–71] to improve prediction performance.

## Data Availability

The data used to support the findings of this study are available from the corresponding author upon request.
